# A comparative study on the characterization of hepatitis B virus quasispecies by clone-based sequencing and third-generation sequencing

**DOI:** 10.1038/emi.2017.88

**Published:** 2017-11-08

**Authors:** Jing Li, Mingjie Wang, Demin Yu, Yue Han, Zhitao Yang, Lei Wang, Xinxin Zhang, Feng Liu

**Affiliations:** 1 Department of Infectious Diseases and Hepatology, The Second Hospital of Shandong University, Jinan, China; 2 Research Laboratory of Clinical Virology, Ruijin Hospital, Shanghai Jiaotong University School of Medicine, Shanghai, China

**Keywords:** genetic heterogeneity, hepatitis B virus, quasispecies, the third-generation sequencing

## Abstract

Hepatitis B virus (HBV) has a high mutation rate due to the extremely high replication rate and the proofreading deficiency during reverse transcription. The generated variants with genetic heterogeneity are described as viral quasispecies (QS). Clone-based sequencing (CBS) is thought to be the ‘gold standard’ for assessing QS complexity and diversity of HBV, but an important issue about CBS is cost-effectiveness and laborious. In this study, we investigated the utility of the third-generation sequencing (TGS) DNA sequencing to characterize genetic heterogeneity of HBV QS and assessed the possible contribution of TGS technology in HBV QS studies. Parallel experiments including 3 control samples, which consisted of HBV full gene genotype B and genotype C plasmids, and 10 patients samples were performed by using CBS and TGS to analyze HBV whole-genome QS. Characterization of QS heterogeneity was conducted by using comprehensive statistical analysis. The results showed that TGS had a high consistency with CBS when measuring the complexity and diversity of QS. In addition, to detect rare variants, there were strong advantages conferred by TGS. In summary, TGS was considered to be practicable in HBV QS studies and it might have a relevant role in the clinical management of HBV infection in the future.

## INTRODUCTION

Hepatitis B virus (HBV) is a hepatotropic virus and its infection is a leading cause of viral hepatitis. Though various prevention measures have contributed to decrease the hepatitis B surface antigen (HBsAg) prevalence in most countries, there still have a stable and increasing HBsAg prevalence in some countries of Africa and Eastern Europe.^1^ HBV is non-cytopathic but can lead to chronic liver inflammation and fibrosis, ultimately result in liver cirrhosis and/or hepatocellular carcinoma.^2^ HBV has a high mutation rate due to the extremely high replication rate and the proofreading deficiency during reverse transcription. The generated variants with genetic heterogeneity are described as viral quasispecies (QS).^3^ Nucleoside/nucleotide analogs are the currently approved antivirals for patients with chronic HBV infection, which target HBV reverse transcriptase (RT) region, thus QS variations within HBV RT region was believed to have an impact on virological response of antiviral treatment.^4^ As documented in several studies, the charcteristics or evolutionary patterns of HBV QS within RT region correlate with the antiviral response to nucleoside/nucleotide analogs and clinical outcomes,^5^ more comprehensive understanding of HBV full-length genome mutations and QS will be helpful to guide the treatment of patients with antiviral treatment.

By so far HBV QS analysis depends on direct PCR sequencing, clone-based sequencing (CBS) and next-generation sequencing. Direct PCR sequencing only detected mutations when mutations reach ~20% of the total HBV QS population.^6^ CBS was considered the ‘gold standard’,^4^ but this method was time-consuming, laborious, low sensitive and incapable to detect novel mutations. Next-generation sequencing has obvious advantages over the previous two methods, but there are limitations, such as the restriction of reading length (maximum 600 bp) and the need to combine the results of molecular cloning.^7^ With the consecutive innovation and application of gene-sequencing technology in recent years, single-molecule real-time sequencing, one of the representatives of the third-generation sequencing (TGS) technology, has been applied in a variety of genome research. Along with the significant increase in reading length,^8^ the new research opportunities have been brought forward to HBV QS. The objective of the present study is to investigate the feasibility and efficacy of TGS on the HBV QS study.

## MATERIALS AND METHODS

### Subjects

Three control samples were manually constructed and a total of 10 patients were enrolled. The recruited patients were admitted to the Department of Infections Disease of the Second Hospital of Shandong University from February 2015 to December 2015. All patients met the clinical diagnostic criteria for chronic hepatitis B^9^ and had discontinued the antiviral treatment at least 1 month before admission. Their serum samples (serum HBV DNA levels >10^5^ copies/mL) were collected at the first day after admission. Individuals with concurrent hepatitis C virus, hepatitis D virus or human immunodeficiency virus infection, alcoholic liver disease, autoimmune liver disease or hepatocellular carcinoma were excluded. Written informed consent was obtained from every patient. The study protocol was approved by the ethics committee of the Second Hospital of Shandong University in accordance with the Declaration of Helsinki. The control samples (C01, C02 and C03) consisted of HBV full gene genotype B and genotype C plasmids, named clone B and clone C, constructed and preserved by the Department of Infections Disease of Ruijin Hospital, Shanghai Jiaotong University School of Medicine. Sample C01 was a mixture of 80% of clone B and 20% of clone C, sample C02 is a mixture of 50% of clone B and 50% of clone C, and sample C03 is a mixture of 20% of clone B and 80% of clone C.

### Liver biochemistry, HBV serology and HBV DNA tests

Liver biochemical analysis was performed with an automated analysis system (Beckman Coulter, Fullerton, CA, USA). HBV serological markers, including HBsAg, antibody to HBsAg, hepatitis B e antigen (HBeAg), antibody to HBeAg and antibody to hepatitis B c antigen were determined by chemiluminescent microparticle enzyme immunoassay (Abbott Laboratories, AbbottPark, IL, USA). HBV DNA levels were tested at a Cobas Amplicor HBV Monitor Test (Roche Diagnostics, Pleasanton, CA, USA), with a lower detection limit of 300 copies/mL.

### DNA extraction and PCR amplification

HBV DNA was extracted from 200 μL of serum samples according to the manufacturer’s instructions (QIAamp DNA Blood MiNi Kit, Qiagen, Hilden, Germany). For CBS, full-length HBV genomes were amplified by PCR based on previously described methods.^10^ Forward primer 5′-TTT TTC ACC TCT GCC TAA TCA-3′ (nucleotides 1821–1841) and reverse primer 5′-AAA AAG TTG CAT GGT GCT GG-3′ (nucleotides 1825–1806) were used. For TGS, samples were amplified with designed barcoded primer pairs (Supplementary Table S1). PCR was performed using KOD-Plus-Neo kit (TOYOBO, Tokyo, Japan) for 40 cycles. The conditions was an initial denaturation at 94 °C for 2 min, and 94 °C for 15 s, 68 °C for 2 min for elongation step, followed by a final extension at 68 °C for 7 min in a thermal cycler. PCR products of full-length HBV genome were purified with a JETQUICK PCR Purification Spin Kit (GENOMED GmbH, Niedersachsen, Germany).

### Clone-based sequencing

PCR-purified products were cloned into PLB-simple Vectors (TIANGEN, Beijing, China) according to the manufacturer’s protocol and subsequently transformed into TOP 10 *Escherichia coli* competent cells (TIANGEN) growing on ampicillin plates. Averages of 30 clones containing the full-length viral genome were sequenced using Applied Biosystems (ABI) 3700 sequencer (Thermo Fisher Scientific, Wilmington, DE, USA). Finally, totals of 105 nucleotide sequences were assembled and were submitted to National Center of Biotechnology Information (NCBI) GenBank under accession numbers KY881721 to KY882003.

### Third-generation sequencing

Purified PCR products were quantified using Qubit 3.0 fluorometer (Thermo Fisher Scientific). PCR products were mixed at final equimolar concentrations of 2 ng/μL. Finally, totals of 7284 nucleotide sequences from 13 samples were submitted to NCBI Sequence Read Archive database (https://www.ncbi.nlm.nih.gov/sra) under the accession number 6650246 to 6650258, which were also included in NCBI BioProject database under the accession number PRJNA380855 (still private, not released yet).

### Sequence manipulation

For CBS, NTI Vector 9 software (Invitrogen, Carlsbad, CA, USA) was used to assemble multiple sequence fragments of the same sample to obtain the whole genome of HBV sequence. Whole-genome sequences were subsequently submitted to HBV STAR site for genotyping.^11^ For TGS, PacBio RSII raw data were first filtered based on quality and read length. Filtered reads were split into each patient according to the barcodes and adapters were then cut to build circular consensus reads. Approximately 500 HBV genome reads per sample were obtained (Supplementary Figure S1).^12^ Open reading frames and functional regions were extracted from the whole-genome sequences, including basic core promoter (*BCP*) gene (nt1742–1849), *C* gene (nt1901–2452), *P* gene (nt2307–3215 and nt1–1623), *PreC* gene (nt1814–1900), *PreS1* gene (nt2848–3204), *PreS2* gene (nt3205–154), *S* gene (nt155–835) and *X* gene (nt1374–1838). All data manipulations were carried out using Perl scripts under version 5.2.0 (Perl Fondation, Walnut, CA, USA, http://www.perl.org/get).

### QS characterization

The viral QS heterogeneity was evaluated at two different levels, including QS complexity and QS diversity, which was also calculated at two different levels, including nucleotide levels and amino-acid levels. The QS complexity, known as Shannon entropy (Sn), the formula: Sn=−Σ_*i*_(*p_i_* In *p_i_*)/In *N*, where *N* is the total number of clones and *p_i_* is the frequency of each clone in the viral QS population.^13^ The QS diversity included the mean genetic distance (*d*), the number of synonymous substitutions per synonymous site (dS), which represents ‘silent’ nucleotide changes that do not alter any amino acid encoded, and the number of non-synonymous substitutions per non-synonymous site (dN), which was calculated for a single sample and represents ‘replacement’ nucleotide changes can result a different amino acid. The mean genetic distance was defined as the number of mutations that distinguished any two sequences from the population. Estimates of average evolutionary divergence over all sequence pairs, the number of amino-acid substitutions per site from averaging over all sequence pairs are shown, using the JTT matrix-based model to analyze.^14^ The rate variation among sites was modeled with a gamma distribution (shape parameter=1). All positions containing gaps and missing data were eliminated, using any positions in the final data set and evolutionary analyses were conducted in MEGA6.^15^ The dS and dN were calculated under the modified NeiGojobori model with Jukes–Cantor correction using MEGA6 software.

### Mutation detection

Compared with the reference of genotype B and genotype C, mutations were detected by two methods. CBS was set as ‘golden standard’, for TGS, 1% mutation frequency was set as the cutoff value, consequently, observing the mutations with a high frequency.

### Phylogenetic analysis

Multiple sequence alignment result in NEXUS format was generated using CLUSTALX 2.1.^16^ Then multiple sequence alignment result was imported into PAUP 4.0[2] to test the nucleotide substitution models using Modeltest 3.7.^17^ Modeltest results showed that GTR+I+G was the best-fit model.^18^ GTR+I+G means General Time Reversible model and Gamma distributed rated and Invariant sites, all the arguments of this model were generated by Modeltest and then imported into PAUP to construct the phylogenetic tree using Maximum likelihood method after bootstrapping to 1000 replicates.

### Statistical analysis

The results of continuous variables were expressed as median and range. The heterogeneity of QS within different regions derived from two methods were calculated, respectively, and compared by using paired Student’s *t*-test or Mann–Whitney test based on the results of Shapiro–Wilks normal distribution test and Bartlett variance homogeneity test. All calculations were conducted by using R 3.3.2 (R Foundation for Statistical Computing, Vienna, Austria) and SPSS 22.0 (IBM, NY, USA). Linear regression models were constructed and correlation analysis of the two methods was carried out using the Pearson correlation test. Bland–Altman was approached to the comparison of the two methods. Because of limited sample size, confidence intervals of the upper and lower bounds of the 95% consistency limits were also indicated in Bland–Altman analysis to avoid relative high sampling error. In all statistical tests, a *P*-value <0.05 was considered statistically significant. Categorical variables between the groups were compared using the *χ*^2^-test. An inter-rater agreement between the tests was also performed (Kappa index: <0=no agreement, 0–0.19=poor, 0.2–0.39=fair, 0.4–0.59=moderate, 0.6–0.79=substantial and 0.8–1.0=almost perfect agreement) to test the consistence between TGS and CBS when referring to the capacity of mutations detection.

## RESULTS

### Demographic, clinical and laboratory data

A total of 10 patients with chronic hepatitis B were enrolled in the present study with a mean age 36.4±7.46 years. In the 8 male and 2 female patients there were 7 HBeAg-positive and 3 HBeAg-negative, with an average viral load of 7.24±0.92 log_10_ copies/ml and an average alanine aminotransferase level of 993.40±887.38 IU/ml. All enrolled patients did not receive antiviral treatment when admission, but they all had history of receiving antiviral treatment, either nucleoside/nucleotide analogs or interferon alfa. (Supplementary Table S2).

### Comparison of QS strains and genotypes obtained with the two methods

An average of 21.77±3.63 clones per sample (total 283 clones) was obtained by CBS. By TGS, ~125 000 raw reads were generated and 560.31±233.42 sequences of HBV full-length genome per sample were finally obtained after filtration and error correction. The number of sequences generated by TGS was significantly larger than those in CBS (*P*<0.01, Student’s *t*-test; Supplementary Table S3).

HBV genotypes identified by using sequences from the two methods matched perfectly. Five out of ten patients were infected with genotypes B strains and the other five patients with genotype C strains (Supplementary Table S2).

### QS composition derived from two methods showed high consistence

In order to compare the heterogeneity determination capacities of CBS and TGS, we structured the control samples, which was composed of HBV full gene clone plasmids of genotype B mixed with genotype C, according to the different proportion divided into C01, C02 and C03 (Table 1). The proportion of C01 is 0.79/0.16 in the CBS and 0.79/0.20 in the TGS, which expected proportion is 0.80/0.20, the proportion of C02 (expected proportion is 0.50/0.50) is 0.47/0.47 in the CBS and 0.50/0.50 in the TGS, and the proportion of C03 (expected proportion is 0.20/0.80) is 0.24/0.71 in the CBS and 0.20/0.79 in the TGS (Table 1). The result showed that the proportion derived from TGS was more approximate to the true value.

**Figure 1:**
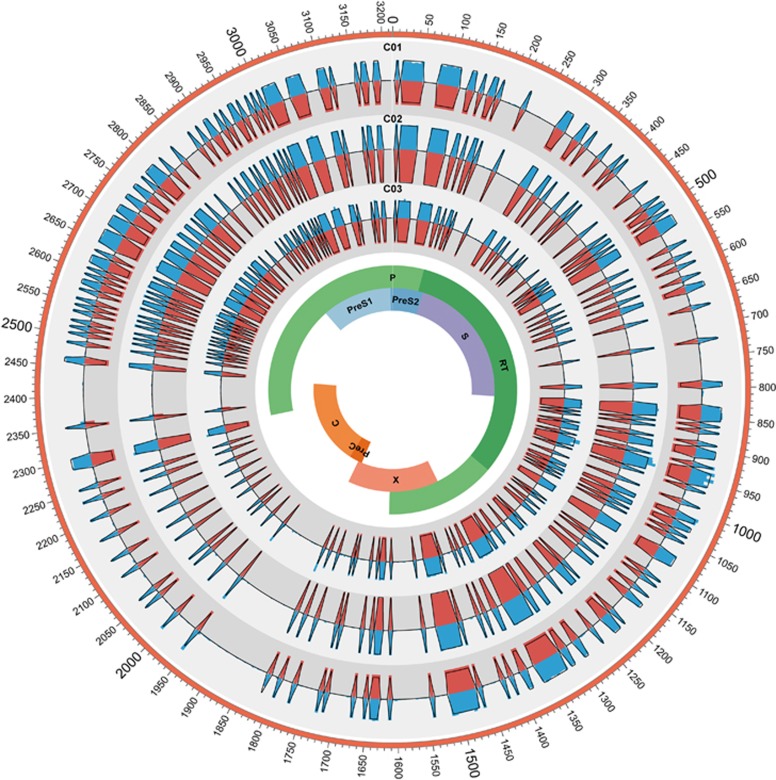
HBV nucleotide acid (nt1–3215) complexity values for C01, C02 and C03. The colored bars indicate the complexity of 5-bp nucleotides calculated by two methods for the full-length HBV genome (blue bars represent TGS and red bars represent CBS). Samples C01, C02 and C03 were presented from outer track to inner track, respectively. Theoretical values of each sample were indicated with black lines. clone-based sequencing, CBS; hepatitis B virus, HBV; third-generation sequencing, TGS.

Furthermore, to thoroughly compare the composition of HBV QS derived from the two methods, the complexity of three control samples were analyzed at single-nucleotide level. As shown in Figure 1, complexity values derived from TGS were highly correlated with those from CBS. In addition, theoretical values of complexities were calculated according to the formula of Shannon entropy and the proportion of the control samples. Both CBS and TGS showed high consistence with their theoretical values. It was noted that the complexity values of TGS were lower than those in CBS, which was due to the calculation formula of Shannon entropy (Sn=−Σ_*i*_(*p_i_* In *p_i_*)/In *N*). As shown in the formula, with the increase of the total number *N*, Sn would reduce gradually even with the same composition of viral QS population (Figure 1).

### Correlation and comparison of QS complexity values derived with the two methods

**Figure 2:**
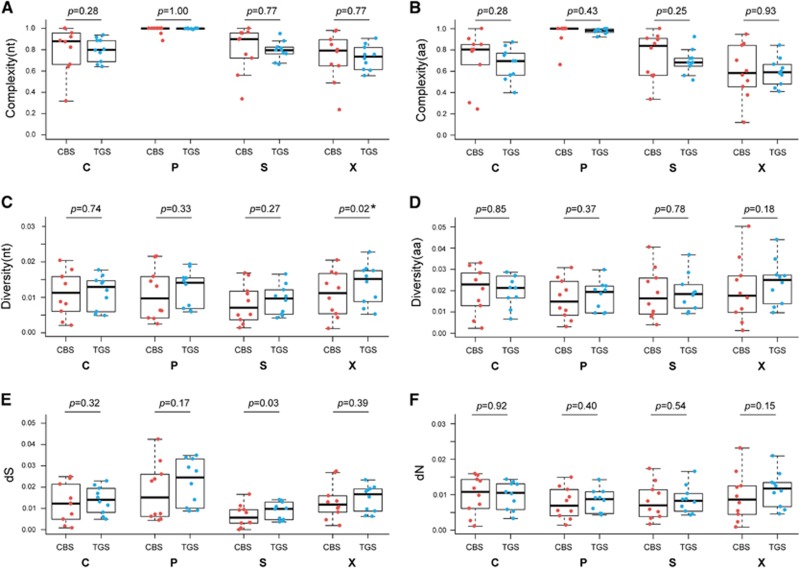
QS complexity at nucleotide level (**A**) and amino-acid level (**B**); genetic distance at nucleotide level (**C**) and amino-acid level (**D**); dS (**E**) and dN (**F**) of *C*, *P*, *S* and *X* ORFs based on CBS and TGS data. Line above the box indicated the maximum, upper line of the box indicated the 75th percentile, line in the box indicated the median value, lower line of the box indicated the 25th percentile and line below the box indicated the minimum. Points in red represent the characteristics of QS derived from CBS, and points in blue represent the characteristics of QS derived from TGS. clone-based sequencing, CBS; open reading frame, ORF; quasispecies, QS; third-generation sequencing, TGS.

In patient samples, at nucleotide level, the QS complexity value derived with TGS showed no difference with that derived with CBS within HBV genome and *BCP* region, *C* region, *P* region, *PreC* region, *PreS1* region, *RT* region, *S* region and *X* region (*P*-value >0.05, Student’s *t*-test or Mann–Whitney test; Supplementary Figure S2). At amino-acid level, the QS complexity value also showed no difference with the two methods within *C* region, *P* region, *PreC* region, RT region, *S* region and *X* region with *P*-value >0.05 (Supplementary Figure S2). However, the QS complexity value derived from TGS was significantly differ from that derived from CBS within PreS1 region (*P*=0.025) and PreS2 region (*P*=0.019; Figure 2). To further compare the correlation of the two methods (Supplementary Figure S3), linear regression models were constructed and correlation efficiencies were calculated. The QS complexity values at nucleotide level and amino-acid level derived with the two methods were highly correlated with *P*-value <0.05 and most of the *R*^2^>0.5 except for whole genome (*P*-value=0.13).

In order to evaluate agreement between the two methods, Bland–Altman analysis was applied, which is the most commonly practiced approach to measuring agreement based on visual observation and to defining limits of agreement.^19, 20^ As indicated by Bland–Altman approach, a high level of agreement was discovered between CBS and TGS except for HBV genome (Supplementary Figure S4).

It is noted that the complexity within HBV PreS2 region, including the nucleotide and amino-acid levels calculated for each patient showed that there was a difference compared with the two methods (*P*-value=0.021 and 0.019 for nucleotide level and amino-acid level, respectively). But on the basis of correlation, CBS was correlated positively with TGS both at nucleotide level and amino-acid level (*R*^2^=0.61, *P*<0.05; *R*^2^=0.45, *P*<0.05, respectively), and on the basis of Bland–Altman analysis, the mean difference between the two methods was −0.14±0.16 (−0.46 to 0.17, lower to upper limit) at the nucleotide level and −0.12±0.13 (−0.36 to 0.13, lower to upper limit) at the amino-acid level, which located within the limits of agreement. Also in *PreS1* region, the complexity from CBS for amino-acid level has a significant difference with the complexity from the TGS by using Student’s *t*-test, however, the correlation showed that the CBS was correlated positively with the TGS and Bland–Altman analysis showed that there was a high level of agreement between CBS and TGS.

### Comparison of QS diversity values derived with the two methods

Both at nucleotide level and amino-acid level, diversity values and dS, dN derived with the two methods had a high consistency, however, at the nucleotide level, the QS distance value derived with the TGS within *BCP* region and *X* region had a significant difference with the genetic distance derived with the CBS (*P*-value=0.044 and 0.021, respectively; Supplementary Figure S2). At *S* region, these two methods differ greatly in dS (*P*=0.030; Figure 2).

Furthermore, the diversity value at nucleotide and amino-acid level, and dS, dN calculated from CBS was highly correlated with that calculated from TGS method for all HBV genome and each partition (Supplementary Figure S3). Bland–Altman analysis demonstrated that there was a high consistency by comparing the two methods for each region including *BCP*, *X* and *S* regions.

### Mutation detection

According to reference D00330 for genotype B and reference AB033556 for genotype C, for CBS sequencing detected a total of 1101 variations over the nucleotides and amino acid of HBV with an average of 110.1±26.98 variations per patient. TGS sequencing detected 3059 variations at an average of 305.9±162.45 variations per patient.

The frequency of common single allele mutation, which had described by several reports,^21, 22, 23, 24^ at RT resign, *S* region, *C* region, *X* region, *Pre C* region and *BCP* region for the two methods in each patient was summarized in Table 2 and Supplementary Table S4. CBS was set as ‘golden standard’, for TGS, 1% mutation frequency was set as the cutoff value. Totally, 50 mutual mutations were detected both by TGS and CBS, reaching a positive rate equaled 15.15%. It was noted that no mutation was detected only by CBS (positive rate=0%). In addition, 40 mutations were detected only by TGS and the positive rate was 12.12%, which was significantly higher than CBS (*χ*^2^=157.14, *P*<0.01). The result showed that TGS had a much higher sensitivity for variation detection than CBS (Table 2, Supplementary Table S5). Furthermore, Kappa consistency test (0.645) demonstrated that the consistency of the two methods was substantial (Supplementary Table S5). For combination mutations, A1762T/G1764A (5/10 patients) and M204[I/V]/A181T (4/10 patients) were detected only by TGS. Combination mutations detected by CBS and TGS at RT resign, BCP region for each patient was summarized in Supplementary Table S6.

### Comparison of QS phylogenetic simulation

**Figure 3:**
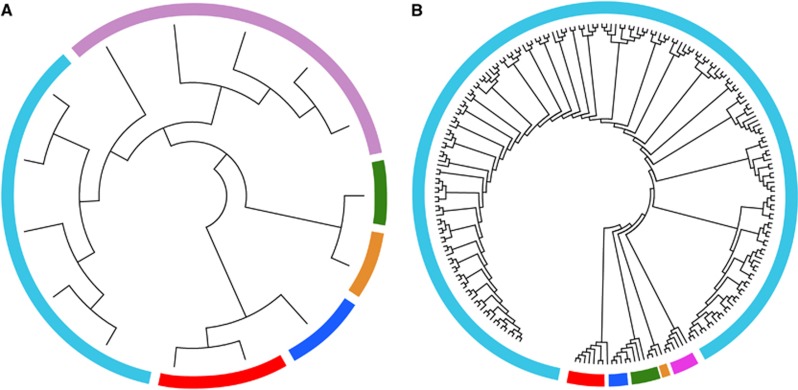
Phylogenetic trees were generated from the full-length HBV genome in patient S08 by CBS (**A**) and TGS (**B**). Strains within different clusters were marked with different colors. clone-based sequencing, CBS; hepatitis B virus, HBV; third-generation sequencing, TGS.

Phylogenetic trees were generated using the full-length HBV genome in patient S08 for demonstration. As shown in Figure 3, homologous virus strains (sequence similarity >0.95) in both trees were marked with the same color and the topology of the two trees was very similar. Besides, phylogenetic tree constructed from TGS data was more complex than that constructed by CBS data. In this study, CBS method produced 25 clones of this sample, while TGS produced up to nearly 220 reads for phylogenetic studies, the evolutionary tree based on TGS data showed more genetic entities than the phylogenetic tree constructed based on CBS.

## Discussion

In the present study, the application of one representative of TGS technology, single-molecule real-time sequencing in HBV QS research was investigated. To compare the genetic heterogeneity of HBV QS measured by CBS and TGS, 3 control samples and 10 clinical samples were collected and various statistical methods were applied for QS characterization. The results revealed that complexity values, diversity values and dS, dN derived from TGS and CBS had a high consistency both at nucleotide level and amino-acid level. Furthermore, in terms of the number of sequences, TGS was superior to CBS and more approximate to the true value. For common mutation, TGS had a much higher sensitivity than CBS.

HBV, as one of double-stranded DNA RT viruses according to Baltimore classification, exists in the form of QS *in vivo*. The composition and evolution of HBV QS were documented to influence the clinical manifestations and prognosis of HBV infection.^25^ As full-length HBV QS may represent the context and the interaction of various genes, it is necessary to study the composition and characteristics of HBV full-length genome QS. CBS is generally considered the ‘gold standard’ for HBV QS research, but this approach is time-consuming in practice and the clone number of full-length genome is usually quite limited. Recent studies reported that the application of the method with PacBio RSII resulted in sequence reads >9 kb that covered the near full-length hepatitis C virus amplicon and enabled the analysis of the near full-length QS,^26^ therefore, it is feasible to apply TGS to HBV full-length genome whose length is about 3000 bp. In the present study, by using different statistical methods, the results indicated TGS had a high consistency with CBS.

This study manifested that TGS had a much higher sensitivity than CBS for common mutations and had a higher consensus accuracy to enable rare variant detection than CBS, especially critical when the ‘minor’ mutant population is of particular clinical significance, such as the RT region, which might lead to nucleoside/nucleotide analog drug resistance during long-term antiviral treatment.^27^ CBS was considered to reliably detect single-nucleotide variants at ≥20% in HBV population,^6^ and TGS had 1% sensitivity and was confirmed with very stable performance for variation detection based on previously published research.^28^ As to the complex interplay of HBV with host immune pressure, antiviral treatment and environmental factors, it was quite necessary to study combination mutations of the virus.^29^ By analyzing the functional combination mutations within BCP and RT regions, it was noted that TGS offered a unique sequencing ability for combination mutations detection and provided more comprehensive information for understanding QS evolution.

As we know, reverse transcription, PCR amplification, CBS and third-generation sequencing all have error-prone steps. PCR can introduce point mutations and indels and generate recombinant sequences, and those variants can be amplificated during PCR, finally, sequencing introduced base substitution errors and indels. For TGS, the error rate was ~15% at single-nucleotide level, but by generating circular consensus reads from single-molecule reads (>3 × 3.2 kb) at threefold coverage, the median accuracy could be lower to 99.6%.^8^ And to distinguish true mutations from technical sequencing errors, 1% mutation frequency was set as the cutoff value, which means that there were at least 2.2 reads harboring the same mutation were considered confidential in TGS, which further increased the accuracy for variation detection.

While the longer read lengths and higher sequencing depth of TGS could overcome plenty of the challenges relating to next-generation sequencing and CBS. However, with the increase of sequencing reads and read length, appropriate bioinformatics analysis tools are desperately needed, such as new types of mathematical models and algorithms to deal with the raw data. With the deeper understanding of genome information, the progress of sequencing technology and the innovation of bioinformatics software, TGS would have a more important role in biological and biomedical research.

As it takes similar time to sequence for both TGS and CBS, TGS is less laborious and time-consuming than CBS, owing to lack of the extra process of cloning into the vectors and transforming into competent cells. Furthermore, TGS costs much less than CBS. In the present study, TGS cost about $250 per sample, while CBS cost about $600 per sample if 30 clones sequenced. Thus TGS has a higher cost-effectiveness.

In summary, the present study indicated the utility of TGS to characterize genetic heterogeneity of HBV QS matches perfectly with CBS. In terms of quantity and the sensitivity of mutation detection, TGS is superior to CBS. With decreases in costs and improvements in sequencing quality, TGS can be used to gain broader insights regarding HBV QS.

## Figures and Tables

**Table 1:** Detection of viral strain compositions in standard mixtures

**Sample**	**Expected proportion**	**Observed composition (CBS)**		**Observed composition (TGS)**	
		**Number of strains (total)**	**Proportion**	**Number of strains (total)**	**Proportion**
C01	0.80/0.20	15/3 (19)	0.79/0.16	552/137 (697)	0.79/0.20
C02	0.50/0.50	9/9 (19)	0.47/0.47	374/370 (747)	0.50/0.50
C03	0.20/0.80	5/15 (21)	0.24/0.71	157/629 (799)	0.20/0.79

Abbreviations: clone-based sequencing, CBS; third-generation sequencing, TGS.

**Table 2:** Number of mutations detected by TGS and CBS

**Region**	**Mutation**	**Positive patients**			
		**TGS+CBS+**	**TGS+CBS−**	**TGS−CBS+**	**TGS−CBS−**
RT	L180M	0	3	0	7
	A181T	0	2	0	8
	M204I	3	2	0	5
	V214A	0	1	0	9
	N236T	0	1	0	9
	M250I	0	2	0	8
S	P120T	1	0	0	9
	T126A	0	1	0	9
	T131N	2	1	0	7
	M133T	0	1	0	9
	D144E	1	0	0	9
	G145R	0	1	0	9
C	L60V	4	2	0	4
	S87G	3	0	0	7
	L100	3	0	0	7
	P130T	3	1	0	6
	P135S	2	1	0	7
	P135Q	1	4	0	5
	P135A	1	1	0	8
X	C1653T	1	0	0	9
	T1674C	0	1	0	9
	T1674G	0	4	0	6
PreC	G1896A	7	2	0	1
	G1899A	2	0	0	8
	G1862T	1	1	0	8
BCP	C1673T	0	1	0	9
	T1753C	2	1	0	7
	A1762T	5	1	0	4
	G1764T	1	0	0	9
	C1766T	2	2	0	6
	T1768A	1	0	0	9
	C1799G	0	1	0	9
	A1846T	4	2	0	4

Abbreviations: basic core promoter, BCP; clone-based sequencing, CBS; reverse transcriptase, RT; third-generation sequencing, TGS.
