# Supplementary material for: A comparative study on the characterization of hepatitis B virus quasispecies by clone-based sequencing and third-generation sequencing
Source: Emerg Microbes Infect. 2017 Nov 8;6(11):e100–. doi: 10.1038/emi.2017.88 (PMC5717089; doi:10.1038/emi.2017.88)
Supplement: Supplementary Table S1 [file emi201788x1.pdf]

Supplementary Table S1. Primer pairs used for HBV full-length genome amplification

Primer Name	Sequence(5'-3')	Strand	Position in HBV genome	Sample Barcode
C01	5'-CGCGCGTGTGTGCGTGTTTTTCA<br>CCTCTGCCTAATCA-3’	Forward	nt1821-nt1841	CGCGCGTG<br>TGTGCGTG
	5'-CACGCACACACGCGCGAAAAAGT<br>TGCATGGTGCTGG-3’	Reverse	nt1825-nt1806	
C02	5'-CACACGCGCGTGCTCGTTTTTCA<br>CCTCTGCCTAATCA-3'	Forward	nt1821-nt1841	CACACGCG<br>CGTGCTCG
	5'-CGAGCACGCGCGTGTGAAAAAGT<br>TGCATGGTGCTGG-3'	Reverse	nt1825-nt1806	
C03	5'-ATCTGTGCGAGACTACTTTTTCAC<br>CTCTGCCTAATCA-3'	Forward	nt1821-nt1841	ATCTGTGC<br>GAGACTAC
	5'-GTAGTCTCGCACAGATAAAAAGTT<br>GCATGGTGCTGG-3'	Reverse	nt1825-nt1806	
S01	5'-ACGCGCACAGAGTCTCTTTTTCAC<br>CTCTGCCTAATCA-3'	Forward	nt1821-nt1841	ACGCGCAC<br>AGAGTCTC
	5'-GAGACTCTGTGCGCGTAAAAAGT<br>TGCATGGTGCTGG-3'	Reverse	nt1825-nt1806	
S02	5'-TCTCTCACAGTCGAGCTTTTTCAC<br>CTCTGCCTAATCA-3'	Forward	nt1821-nt1841	TCTCTCACA<br>GTCGAGC
	5'-GCTCGACTGTGAGAGAAAAAAGT<br>TGCATGGTGCTGG-3'	Reverse	nt1825-nt1806	
S03	5'-GTCATCACACATCTCTTTTTTCAC<br>CTCTGCCTAATCA-3'	Forward	nt1821-nt1841	GTCATCAC<br>ACATCTCT
	5'-AGAGATGTGTGATGACAAAAAGTT<br>GCATGGTGCTGG-3'	Reverse	nt1825-nt1806	
S04	5'-CTGATATGTAGTCGTATTTTTCAC<br>CTCTGCCTAATCA-3'	Forward	nt1821-nt1841	CTGATATGT<br>AGTCGTA
	5'-TACGACTACATATCAGAAAAAGTT<br>GCATGGTGCTGG-3'	Reverse	nt1825-nt1806	
S05	5'-AGACTCTACAGAGATATTTTTCAC<br>CTCTGCCTAATCA-3'	Forward	nt1821-nt1841	AGACTCTA<br>CAGAGATA
	5'-TATCTCTGTAGAGTCTAAAAAGTT<br>GCATGGTGCTGG-3'	Reverse	nt1825-nt1806	
S06	5'-GCGCATGTCTCTCTCTTTTTTCAC<br>CTCTGCCTAATCA-3'	Forward	nt1821-nt1841	GCGCATGT<br>CTCTCTCT
	5'-AGAGAGAGACATGCGCAAAAAGT<br>TGCATGGTGCTGG-3'	Reverse	nt1825-nt1806	
S07	5'-TCTACAGAGCGAGAGTTTTTTCAC<br>CTCTGCCTAATCA-3'	Forward	nt1821-nt1841	TCTACAGA<br>GCGAGAGT
	5'-ACTCTCGCTCTGTAGAAAAAAGTT	Reverse	nt1825-nt1806	

	GCATGGTGCTGG-3'			
S08	5'-ACTACTGAGACATAGATTTTTCAC CTCTGCCTAATCA-3'	Forward	nt1821-nt1841	ACTACTGA GACATAGA
	5'-TCTATGTCTCAGTAGTAAAAAGTT GCATGGTGCTGG-3'	Reverse	nt1825-nt1806	
S09	5'-CGCATGAGATATACGCTTTTTCAC CTCTGCCTAATCA-3'	Forward	nt1821-nt1841	CGCATGAG ATATACGC
	5'-GCGTATATCTCATGCGAAAAAGTT GCATGGTGCTGG-3'	Reverse	nt1825-nt1806	
S10	5'-GTAGCGACATACGCACTTTTTCAC CTCTGCCTAATCA-3'	Forward	nt1821-nt1841	GTAGCGAC ATACGCAC
	5'-GTGCGTATGTCGCTACAAAAAGTT GCATGGTGCTGG-3'	Reverse	nt1825-nt1806	
