# Supplementary material for: A comparative study on the characterization of hepatitis B virus quasispecies by clone-based sequencing and third-generation sequencing
Source: Emerg Microbes Infect. 2017 Nov 8;6(11):e100–. doi: 10.1038/emi.2017.88 (PMC5717089; doi:10.1038/emi.2017.88)
Supplement: Supplementary Table S2 [file emi201788x2.pdf]

Supplementary Table S2. General information of enrolled patients

Patient ID	Gender	Age（years）	HBV DNA (log10 copies/mL)	Genotype	ALT	HBeAg status	Treatment	Treatment duration
S01	M	29	5.91	B	3287.2	+	ETV	2014.03-2014.12
S02	M	39	6.34	C	224.8	+	ETV	2014.1-2014.8
S03	M	46	8.23	B	304	-	LAM+ADV	2012.09.17-2015.11
S04	M	43	7.50	B	1052	+	IFN	2010-2014
S05	M	35	6.66	C	792	+	IFN	2009.01-2009.07
S06	M	28	7.05	B	1293	+	ETV	2005-2011
S07	M	40	7.26	C	332	+	LAM<br>ADV	2009-2011<br>2011-2014.08
S08	M	23	8.18	B	1150	+	IFN	2012.06-2013.12
S09	F	42	8.75	C	899	-	LAM	2012-2014.06
S10	F	39	6.55	C	600	-	LDT<br>ADV	2009.11-2011.03<br>2010.08-2011.03
Mean ±SD	8/2	36.4 ±7.46	7.24 ±0.92	5/5	993.40 ±887.38	7/3		

Abbreviations: +, positive; -, negative; ETV, Entecavir; LAM, Lamivudine; ADV, Adefovir Dipivoxil; IFN, Interferon alfa; LDT, Telbivudine
