# Supplementary material for: A comparative study on the characterization of hepatitis B virus quasispecies by clone-based sequencing and third-generation sequencing
Source: Emerg Microbes Infect. 2017 Nov 8;6(11):e100–. doi: 10.1038/emi.2017.88 (PMC5717089; doi:10.1038/emi.2017.88)
Supplement: Supplementary Table S3 [file emi201788x3.pdf]

Supplementary Table S3. Number of sequences generated by CBS and TGS

Sample	Number of sequences (CBS)	Number of sequences (TGS)
C01	19	697
C02	19	747
C03	21	799
S01	22	459
S02	18	378
S03	24	222
S04	26	245
S05	23	780
S06	22	563
S07	29	816
S08	15	220
S09	21	798
S10	24	560
