# Supplementary material for: A comparative study on the characterization of hepatitis B virus quasispecies by clone-based sequencing and third-generation sequencing
Source: Emerg Microbes Infect. 2017 Nov 8;6(11):e100–. doi: 10.1038/emi.2017.88 (PMC5717089; doi:10.1038/emi.2017.88)
Supplement: Supplementary Table S4 [file emi201788x4.pdf]

Supplementary Table S4. Mutation profiles derived from CBS and TGS for each patient (S01-S05)

Region	Mutation	S01		S02		S03		S04		S05	
		CBS	TGS	CBS	TGS	CBS	TGS	CBS	TGS	CBS	TGS
RT	L180M	-	-	-	-	-	-	-	-	-	-
	A181T	-	-	-	0.0185	-	-	-	-	-	0.0128
	M204I	-	-	-	-	-	0.0179	-	0.0407	-	-
	V214A	-	-	-	0.0237	-	-	-	-	-	-
	N236T	-	-	-	-	-	0.0448	-	-	-	-
	M250I	-	-	-	-	-	0.0269	-	0.0163	-	-
S	P120T	-	-	0.3158	0.2269	-	-	-	-	-	-
	T126A	-	-	-	-	-	0.0628	-	-	-	-
	T131N	-	-	-	-	-	-	-	-	0.125	0.0602
	M133T	-	-	-	-	-	-	-	-	-	-
	D144E	-	-	0.3684	0.3905	-	-	-	-	-	-
	G145R	-	-	-	0.0396	-	-	-	-	-	-
C	L60V	0.913	0.9239	-	0.0106	0.92	0.9327	0.8519	0.9512	-	-
	S87G	-	-	-	-	-	-	-	-	-	-
	L100	-	-	-	-	-	-	-	-	-	-
	P130T	-	-	-	-	-	-	-	-	0.8333	0.7977
	P135S	-	-	0.1579	0.0923	-	-	0.2222	0.2764	-	-
	P135Q	-	0.0826	-	0.058	-	0.1121	-	0.0285	-	-
	P135A	-	-	-	0.0106	0.68	0.6502	-	-	-	-
X	C1653T	-	-	0.2105	0.2137	-	-	-	-	-	-
	T1674C	-	-	-	-	-	-	-	-	-	0.0102
	T1674G	-	-	-	0.0343	-	-	-	-	-	0.0141
PreC	G1862T	-	-	-	-	-	-	0.0741	0.0569	-	0.0569
	G1896A	-	0.0174	0.1579	0.2322	0.92	0.9372	0.8519	0.9065	-	0.9065
	G1899A	-	-	-	-	-	-	0.8148	0.9593	-	0.9593
BCP	C1673T	-	-	-	0.0422	-	-	-	-	-	-
	T1753C	-	-	-	-	-	-	-	-	0.2083	0.1204
	A1762T	0.9565	0.9478	0.2632	0.2322	0.68	0.8027	0.2222	0.2724	-	-
	G1764T	-	-	0.2105	0.0844	-	-	-	-	-	-
	C1766T	-	-	-	-	0.08	0.0942	-	-	0.0833	0.0691
	T1768A	-	-	-	-	0.08	0.0807	-	-	-	-
	C1799G	-	-	-	0.029	-	-	-	-	-	-
	A1846T	0.0870	0.0217	-	0.0132	0.92	0.9372	0.9259	0.9634	-	-

Mutations detected by TGS and not detected by CBS were highlighted in blue background.

Mutations not detected in patient samples were shown in hypen

Supplementary Table S4 (cont'd) Mutation profiles derived from CBS and TGS for each patient (S06-S10)

Region	Mutation	S06		S07		S08		S09		S10	
		CBS	TGS	CBS	TGS	CBS	TGS	CBS	TGS	CBS	TGS
RT	L180M	-	-	-	0.0196	-	-	-	0.01	-	0.0143
	A181T	-	-	-	-	-	-	-	-	-	-
	M204I	-	-	0.9667	0.9718	-	-	0.5000	0.4981	0.4	0.5651
	V214A	-	-	-	-	-	-	-	-	-	-
	N236T	-	-	-	-	-	-	-	-	-	-
	M250I	-	-	-	-	-	-	-	-	-	-
S	P120T	-	-	-	-	-	-	-	-	-	-
	T126A	-	-	-	-	-	-	-	-	-	-
	T131N	-	-	-	-	-	-	-	0.0263	0.08	0.0232
	M133T	-	-	-	-	-	0.0226	-	-	-	-
	D144E	-	-	-	-	-	-	-	-	-	-
	G145R	-	-	-	-	-	-	-	-	-	-
C	L60V	0.9565	0.9823	-	-	-	0.0131	-	-	-	-
	S87G	-	-	0.9667	0.9767	-	-	0.5000	0.5019	0.4000	0.5668
	L100	-	-	0.7000	0.7087	-	-	0.3636	0.3617	0.3600	0.4171
	P130T	-	-	-	-	-	0.0136	0.4091	0.3942	0.4400	0.3369
	P135S	-	0.0106	-	-	-	-	-	-	-	-
	P135Q	-	-	-	-	0.3125	0.1991	-	-	-	-
	P135A	-	-	-	-	-	-	-	-	-	-
X	C1653T	-	-	-	-	-	-	-	-	-	-
	T1674C	-	-	-	-	-	-	-	-	-	-
	T1674G	-	-	-	-	-	0.0181	-	0.0113	-	-
PreC	G1862T	-	0.0957	-	-	-	-	-	-	-	-
	G1896A	0.9130	0.9131	0.1667	0.2644	0.9375	0.9593	0.0909	0.1314	-	0.1569
	G1899A	0.9565	0.9734	-	-	-	-	-	-	-	-
BCP	C1673T	-	-	-	-	-	-	-	-	-	-
	T1753C	-	-	-	-	-	-	-	0.0613	0.0800	0.0553
	A1762T	-	0.0142	-	-	0.3750	0.4751	-	-	-	-
	G1764T	-	-	-	-	-	-	-	-	-	-
	C1766T	-	-	-	-	-	-	-	0.0375	-	0.0321
	T1768A	-	-	-	-	-	-	-	-	-	-
	C1799G	-	-	-	-	-	-	-	-	-	-
	A1846T	0.9565	0.9787	-	-	-	-	-	-	-	0.0107

Mutations detected by TGS and not detected by CBS were highlighted in blue background.
Mutations not detected in patient samples were shown in hypen
