# Supplementary material for: A comparative study on the characterization of hepatitis B virus quasispecies by clone-based sequencing and third-generation sequencing
Source: Emerg Microbes Infect. 2017 Nov 8;6(11):e100–. doi: 10.1038/emi.2017.88 (PMC5717089; doi:10.1038/emi.2017.88)
Supplement: Supplementary Table S5 [file emi201788x5.pdf]

Supplementary Table S5. Comparison of two methods for detection of mutations

TGS	CBS		Total
	+	-	
+	50	40	90
-	0	240	240
Total	50	280	330

+:positive; -:negtive
