# Supplementary material for: A comparative study on the characterization of hepatitis B virus quasispecies by clone-based sequencing and third-generation sequencing
Source: Emerg Microbes Infect. 2017 Nov 8;6(11):e100–. doi: 10.1038/emi.2017.88 (PMC5717089; doi:10.1038/emi.2017.88)
Supplement: Supplementary Table S6 [file emi201788x6.pdf]

Supplementary Table S6. Combination mutations detected by CBS and TGS for each patient (S01-S05).

Region	Combination Mutation	S01		S02		S03		S04		S05	
		CBS	TGS	CBS	TGS	CBS	TGS	CBS	TGS	CBS	TGS
BCP	A1762T/G1764A	1.0000	0.9434	0.2778	0.2222	0.6667	0.7748	0.2308	0.2694	-	0.0051
	G1764[A/T]/C1766T	-	0.0022	0.0556	0.0053	-	0.0270	-	-	0.0870	0.0692
	C1766T/T1768A	-	-	-	-	0.0833	0.0856	-	-	-	-
RT	M204[I/V]/A181T	-	-	0.0556	0.0053	-	-	-	0.0082	-	0.0026
	N236K/A181T	-	-	-	0.0026	-	-	-	-	-	-

Combination Mutations detected by TGS and not detected by CBS were highlighted in blue background.

Combination Mutations not detected in patient samples were shown in hypen

Supplementary Table S6 (cont′d). Combination mutations detected by CBS and TGS for each patient (S06-S10).

Region	Combination Mutation	S06		S07		S08		S09		S10	
		CBS	TGS	CBS	TGS	CBS	TGS	CBS	TGS	CBS	TGS
BCP	A1762T/G1764A	-	0.0142	-	0.0037	0.4000	0.4227	-	0.0025	-	0.0036
	G1764[A/T]/C1766T	-	-	-	-	-	0.0045	0.0476	0.0376	0.0417	0.0321
	C1766T/T1768A	-	0.0018	-	-	-	0.0045	-	-	-	-
RT	M204[I/V]/A181T	-	-	-	0.0012	-	-	-	-	-	0.0018
	N236K/A181T	-	-	-	-	-	-	-	-	-	-

Combination Mutations detected by TGS and not detected by CBS were highlighted in blue background.

Combination Mutations not detected in patient samples were shown in hypen
