# Supplementary figures and images for: A comparative study on the characterization of hepatitis B virus quasispecies by clone-based sequencing and third-generation sequencing
Source: Emerg Microbes Infect. 2017 Nov 8;6(11):e100–. doi: 10.1038/emi.2017.88 (PMC5717089; doi:10.1038/emi.2017.88)

**Supplementary Figure S1:** Sequence manipulation pipeline for the TGS data.

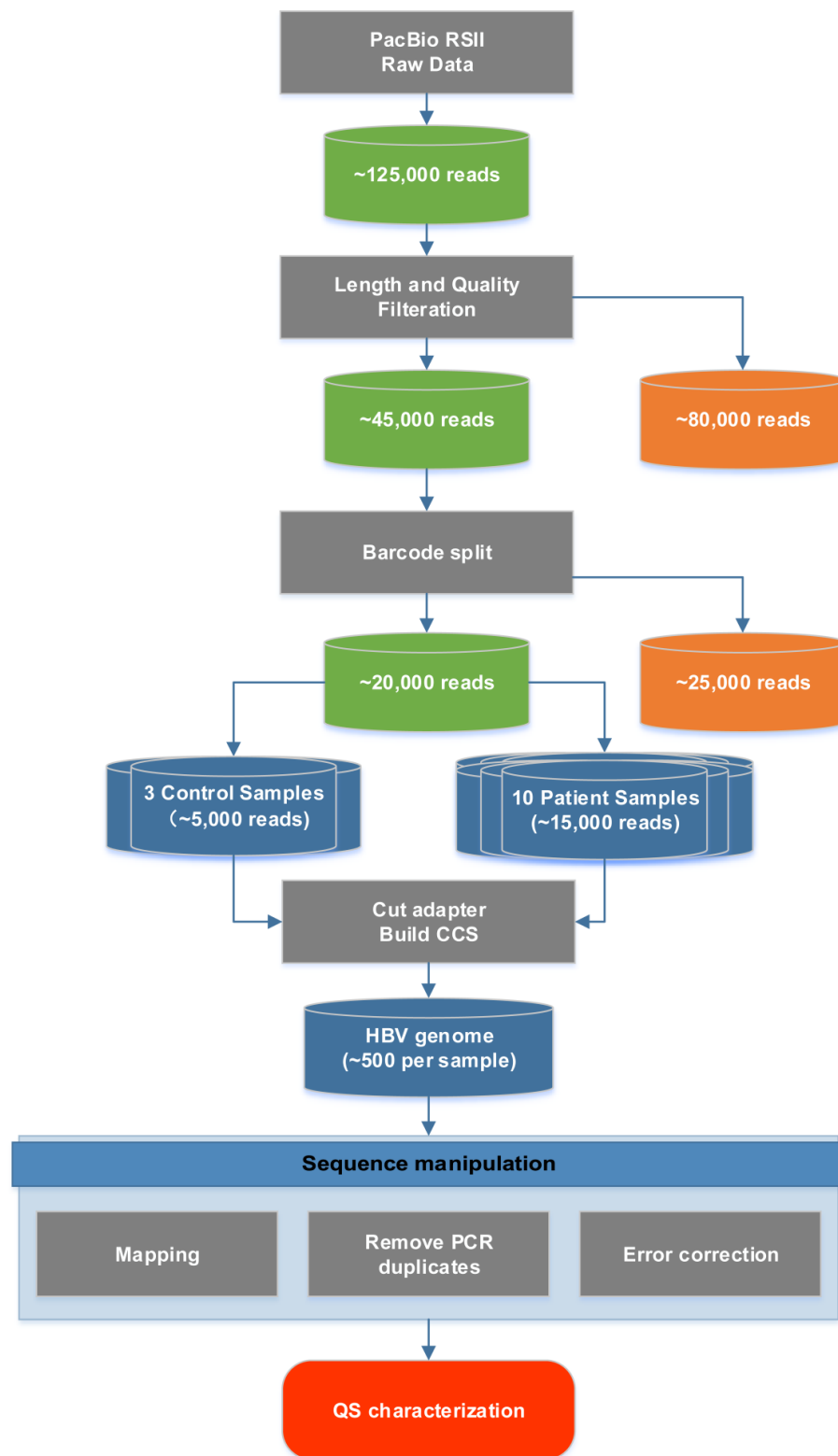

Supplement: Supplementary Figure S1 [file emi201788x7.pdf]
