# Supplementary material for: A comparative study on the characterization of hepatitis B virus quasispecies by clone-based sequencing and third-generation sequencing
Source: Emerg Microbes Infect. 2017 Nov 8;6(11):e100–. doi: 10.1038/emi.2017.88 (PMC5717089; doi:10.1038/emi.2017.88)

Supplementary Figure S2: HBV whole genome, BCP, C, P, PreC, PreS1, PreS2, RT, S, X QS at nucleotide level (A) and amino acid level (B), genetic distance at nucleotide level (C) and amino acid level (D), dS (E) and dN (F) based on CBS and TGS data. Red points represent the characteristics of QS of each patient for CBS. Blue points represent the characteristics of QS of each patient for TGS. (*, *P*<0.05)

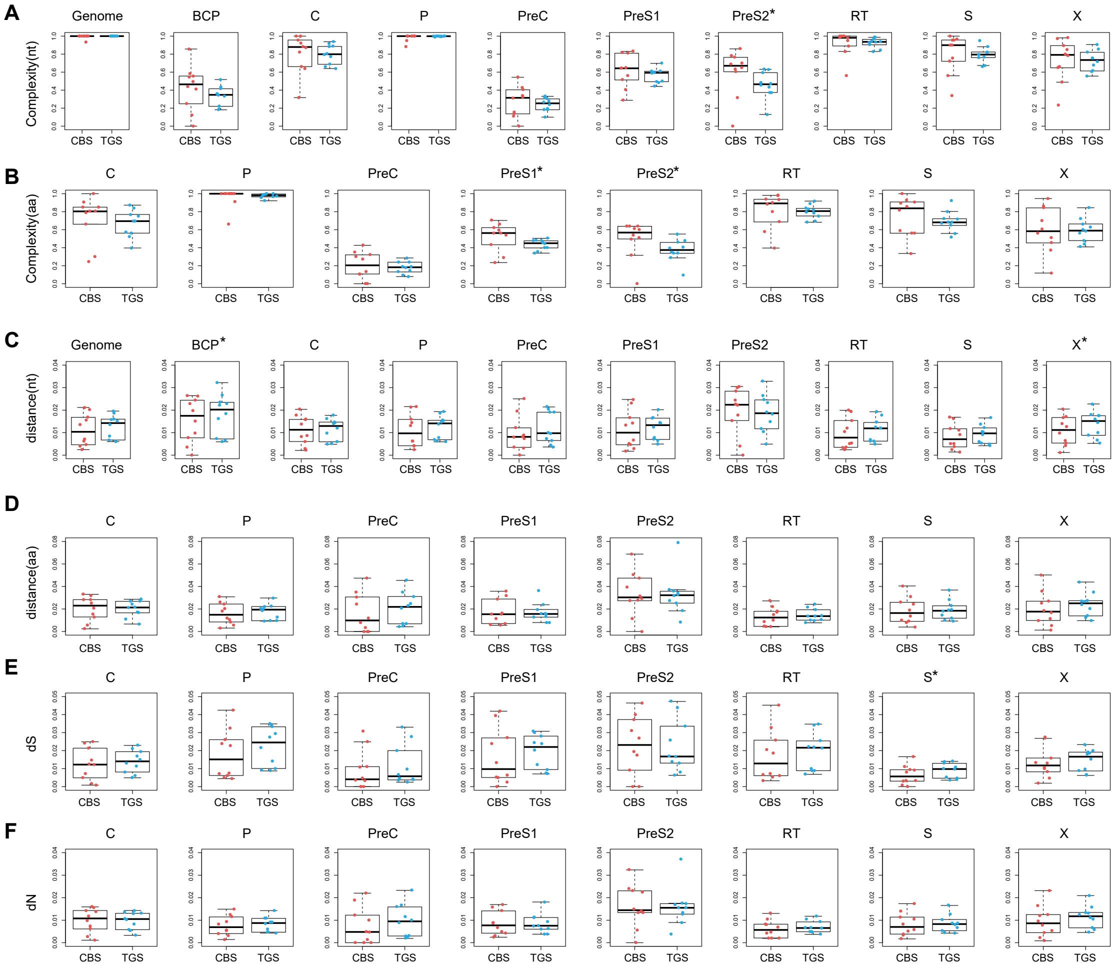

Supplement: Supplementary Figure S2 [file emi201788x8.pdf]
