# Supplementary material for: A comparative study on the characterization of hepatitis B virus quasispecies by clone-based sequencing and third-generation sequencing
Source: Emerg Microbes Infect. 2017 Nov 8;6(11):e100–. doi: 10.1038/emi.2017.88 (PMC5717089; doi:10.1038/emi.2017.88)

Supplementary Figure S3: Scatter plot and line regression of HBV whole genome, BCP, C, P, PreC, PreS1, PreS2, RT, S, X QS complexity at nucleotide level (A) and amino acid level (B), genetic distance at nucleotide level (C) and amino acid level (D), dS (E) and dN (F) based on CBS and TGS data. P and R^2 values of line regression analysis are indicated in the plots. (*, $P < 0.05$)

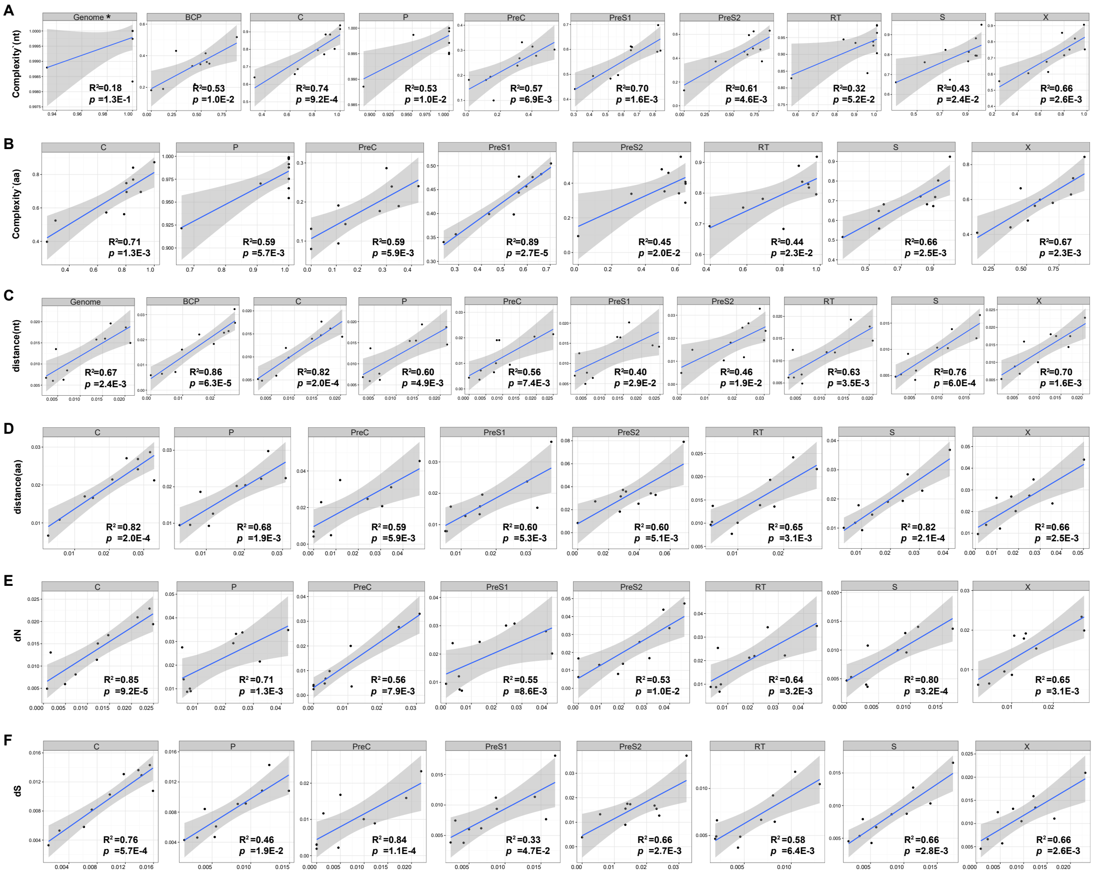

Supplement: Supplementary Figure S3 [file emi201788x9.pdf]
