# Supplementary material for: A comparative study on the characterization of hepatitis B virus quasispecies by clone-based sequencing and third-generation sequencing
Source: Emerg Microbes Infect. 2017 Nov 8;6(11):e100–. doi: 10.1038/emi.2017.88 (PMC5717089; doi:10.1038/emi.2017.88)

Supplementary Figure S4: Bland-Altman analysis for HBV whole genome, BCP, C, P, PreC, PreS1, PreS2, RT, S, X QS complexity at nucleotide level (A) and amino acid level (B), genetic distance at nucleotide level (C) and amino acid level (D), dS (E) and dN (F) based on CBS and TGS data. The blue horizontal lines were drawn at the mean difference; the red horizontal lines were drawn at the upper and lower limits of agreement, which were defined as the mean difference ± 1.96×SD of the difference. The short vertical lines indicate the 95% consistency limit of upper and lower bounds of agreement. Values within the limits are shown in blue dots. Values exceed the limits are shown in red and marked with arrows.

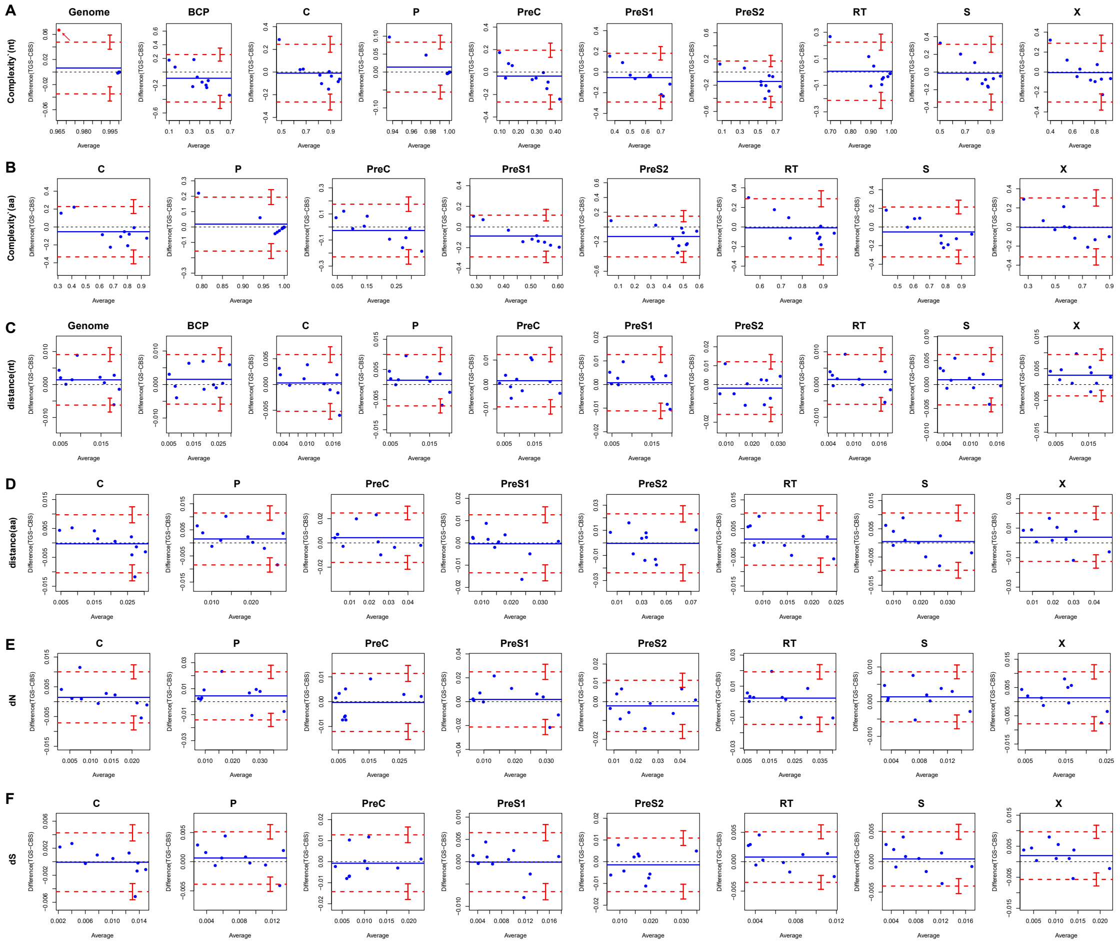

Supplement: Supplementary Figure S4 [file emi201788x10.pdf]
